# Transurethral 1470 nm diode laser vaporization versus plasma kinetic enucleation of the prostate for the treatment of benign prostatic hyperplasia: A retrospective study

**DOI:** 10.1097/MD.0000000000035031

**Published:** 2023-08-25

**Authors:** Jiaguo Huang, Yi Fan, Kai Wang, Hongxiang Ding, Dikai Mao, Liwei Zhao, Shengcheng Tai

**Affiliations:** a Department of Urology, Affiliated Xiaoshan Hospital, Hangzhou Normal University, Hangzhou, Zhejiang, China; b Department of Urology, Affiliated Hangzhou First People’s Hospital, Zhejiang University School of Medicine, Hangzhou, Zhejiang, China.

**Keywords:** 1470 nm diode laser vaporization, benign prostatic hyperplasia, clinical efficacy, plasma kinetic enucleation, retrospective study

## Abstract

To compare the efficacy, safety, and complications of transurethral 1470 nm diode laser vaporization and plasma kinetic enucleation of prostate (PKEP) in benign prostatic hyperplasia treatment. A retrospective matched-paired comparison of patients treated using transurethral 1470 nm diode laser vaporization (n = 40) or PKEP (n = 40) was conducted. Baseline characteristics, preoperative data, and postoperative outcomes at the 24-month follow-up of the patients were recorded. The present study found no significant preoperative differences between the 2 treatment groups. Compared with PKEP, 1470 nm diode laser vaporization had a significantly shorter operation time and less intraoperative blood loss, but there were no marked differences between the 2 groups in terms of postoperative bladder irrigation time, catheterization time, and hospital stay. Moreover, at the 24-month follow-up postoperatively, there were no marked differences in the International Prostatic Symptomatic Score (IPSS), quality of life (QOL), maximum urinary flow rate (Qmax), and post-void residual urine volume (PVR) between the 2 groups. IPSS, QOL, Qmax, and PVR had improved significantly compared to preoperative assessment at 24-month follow-up in both groups and there was no significant difference in the variation of IPSS, QOL, Qmax and PVR before and after the operation. Furthermore, complications were comparable between the 2 treatment groups. Transurethral 1470 nm diode laser vaporization and PKEP are effective strategies in the treatment of benign prostatic hyperplasia. However, 1470 nm diode laser vaporization offers advantages over PKEP in terms of shortening operation time and reducing intraoperative bleeding. Nonetheless, further research with a larger number of patients and long-term follow-up is necessary to confirm and validate these findings.

## 1. Introduction

Benign prostatic hyperplasia (BPH) is one of the most prevalent conditions affecting men. Currently, transurethral resection of the prostate (TURP) is considered the gold standard for the surgical treatment of BPH. TURP can greatly alleviate lower urinary tract symptoms and dysuria, and is the most widely used technique clinically.^[1–4]^ With the advancement of minimally invasive technology, laser, and plasma methods are increasingly being used in prostate surgery. Interestingly, a randomized controlled study demonstrated that plasma kinetic enucleation of the prostate (PKEP) outperformed TURP in terms of bleeding, postoperative bladder irrigation time, catheterization time, and hospital stay.^[5]^ In recent years, various laser technologies have been gradually applied to the clinical treatment of BPH due to their shorter operation time, less intraoperative bleeding, fewer postoperative complications, and better safety profile.^[6]^ Commonly used lasers include holmium laser, green laser, and thulium laser.^[7–10]^ Some studies have suggested that 980 nm diode laser vaporization has the same curative effect as TURP, but it has a higher hemostatic potential.^[11–14]^ However, there are few reports on the use of 1470 nm diode laser vaporization. Additionally, there is no existing research comparing the therapeutic effect and safety between 1470 nm diode laser vaporization and PKEP. To that end, the present study aims to retrospectively compare the efficacy and safety of the 2 interventions for BPH treatment.

## 2. Material and methods

### 2.1. Case selection

A retrospective matched-paired comparison study was conducted to compare the outcomes of transurethral 1470 nm diode laser vaporization and PKEP. The study was approved by the Ethical Committee of Affiliated Xiaoshan Hospital, Hangzhou Normal University. The study included a total of 117 patients who underwent either transurethral 1470 nm diode laser vaporization or PKEP between June 2018 and June 2020 at a single center. The selection of patients adhered to the indications and contraindications for treating BPH as specified in the Chinese guidelines.^[15]^ The inclusion criteria were as follows: a maximum urinary flow rate (Qmax) of ≤ 15 mL/seconds and an International Prostate Symptom Score (IPSS) of ≥ 7. Patients with suspected or known prostate cancer or neurogenic bladder, as well as those that underwent previous prostatic or urethral surgery, were excluded from the study. Preoperative assessment included a digital rectal examination, transabdominal B-ultrasound, serum prostate-specific antigen, IPSS, quality of life (QOL), Qmax and post-void residual urine volume (PVR). Patients with diabetes, hypertension, or heart conditions were treated accordingly prior to the interventions in order to meet the surgical requirements. Before undergoing surgery, all patients were thoroughly informed about the potential risks and complications associated with the procedure and anesthesia. They were also required to sign an informed consent form, providing approval for the use of their data for research purposes.

### 2.2. Postoperative follow-up

The surgery time, intraoperative blood loss, postoperative bladder irrigation time, catheterization time, hospital stay were recorded. We estimated intraoperative blood loss by the variation of preoperative and postoperative hemoglobin levels. The PVR, Qmax, IPSS and QOL of the 2 groups were collected at 24 months after the procedures. Postoperative complications, including urinary tract infection, need for blood transfusion, urethral stricture, and urinary retention, were also recorded during follow-ups.

### 2.3. Statistical methods

Statistical analyses were performed using Statistical Package for the Social Sciences (SPSS 23.0, SPSS Inc., Chicago, IL). Continuous variables are presented as mean ± standard deviation, while categorical data are reported as number of cases (percentage of cases). Normally distributed parameters data were analyzed using the Student *t* test. Meanwhile, for non-normally distributed data, a Wilcoxon rank sum test was used. Categorical data were compared using the chi-square test or Fisher exact probability test. *P* values < .05 were considered statistically significant.^[16]^

## 3. Results

In total, 80 patients who met the inclusion and exclusion criteria were included in the study. Among them, 40 patients underwent transurethral 1470 nm diode laser vaporization, and they were matched with another 40 patients who underwent PKEP. The baseline characteristics and preoperative data of the patients are presented in Table 1. There were no significant differences in preoperative baseline data, including age, prostate volume, serum prostate-specific antigen level, IPSS, QOL, Qmax, and PVR between the 2 groups (*P* < .05).

**Table 1:** Baseline patient characteristics.

	1470 nm laser group (n = 40)	PKEP group (n = 40)	*P* value
Age (yr)	70.40 ± 8.04	70.70 ± 7.14	.86
Prostate volume (mL^3^)	51.70 ± 23.45	51.03 ± 33.11	.158
PSA (ng/mL)	4.37 ± 4.67	5.70 ± 4.85	.131
IPSS	28.73 ± 4.10	29.75 ± 3.26	.361
QOL	5.00 ± 0.85	4.75 ± 1.03	.073
Qmax (mL/s)	6.95 ± 3.25	7.80 ± 3.24	.245
PVR (mL)	126.33 ± 152.02	146.00 ± 132.03	.626

IPSS = International Prostatic Symptomatic Score, PKEP = plasma kinetic enucleation of prostate, PSA = prostate-specific antigen, PVR = post-void residual urine volume, Qmax = maximum urinary flow rate, QOL = quality of life.

All patients successfully underwent their respective operations. Compared with the PKEP group, the 1470 nm laser group had a significantly shorter surgery time and less intraoperative blood loss, as evidenced by the small variation between preoperative and postoperative hemoglobin levels (*P* > .05, Table 2). However, there were no significant differences between the 2 groups in terms of postoperative bladder irrigation time, catheterization time, and hospital stay (*P* < .05, Table 2). At the 2-year follow-up after the operation, there were no significant differences between the 2 groups in terms of IPSS, QOL, Qmax, and PVR (*P* > .05, Table 2). Notably, both groups showed significant improvements in IPSS, QOL, Qmax, and PVR compared to their respective preoperative assessments (Table 3). Moreover, there were no significant differences in the variation of IPSS, QOL, Qmax and PVR before and after operation in both groups (*P* > .05, Table 3).

**Table 2:** Preoperative and postoperative outcomes in both treatment groups.

	1470 nm laser group (n = 40)	PKEP group (n = 40)	*P* value
Surgery time (min)	62.23 ± 26.51	90.35 ± 43.06	<.001
Variation of hemoglobin (g/L)	9.43 ± 7.56	12.90 ± 7.57	.027
Bladder irrigation (d)	2.08 ± 0.97	2.03 ± 0.95	.816
Catheterization time (d)	6.33 ± 1.29	6.73 ± 1.83	.261
Hospital Stay (d)	8.65 ± 1.23	9.15 ± 3.02	.992
Postoperative IPSS	3.45 ± 1.45	3.38 ± 1.31	.809
Postoperative QOL	1.68 ± 0.83	1.35 ± 0.92	.101
Postoperative Qmax (mL/s)	16.33 ± 2.52	16.88 ± 2.20	.301
Postoperative PVR (mL)	10.75 ± 15.99	7.15 ± 22.00	.054

IPSS = International Prostatic Symptomatic Score, PKEP = plasma kinetic enucleation of prostate, PVR = post-void residual urine volume, Qmax = maximum urinary flow rate, QOL = quality of life.

**Table 3:** Preoperative and postoperative values variation in both treatment groups.

	1470 nm laser group (n = 40)	PKEP group (n = 40)	*P* value
Variation of IPSS	25.28 ± 3.25	26.38 ± 3.40	.143
Variation of QOL	3.18 ± 0.93	3.40 ± 1.46	.415
Variation of Qmax (mL/s)	9.38 ± 4.16	9.08 ± 3.60	.640
Variation of PVR (mL)	115.58 ± 147.02	138.85 ± 128.65	.396

IPSS = International Prostatic Symptomatic Score, PKEP = plasma kinetic enucleation of prostate, PVR = post-void residual urine volume, Qmax = maximum urinary flow rate, QOL = quality of life.

During the 24-month follow-up period, all complications experienced by the patients in both groups were documented in Table 4. In the 1470 nm laser group, there were no cases of urinary incontinence reported; but 1 patient developed high fever 48 hours postoperatively, another patient experienced hematuria 1 week after the intervention, which improved after receiving a blood transfusion, and 1 patient developed urethral stricture 3 months after the procedure. In the PKEP group, 3 patients experienced significant bleeding postoperatively after returning to the ward and required a blood transfusion. Among them, 1 patient needed to return to the operating room to control the bleeding, while the other 2 patients showed improvement with conservative treatment. Additionally, 2 patients in the PKEP group had postoperative urinary tract infections, and 2 cases (5%) had slightly impaired urinary control and experienced urinary incontinence after extubation. These patients successfully recovered after 1 month of Kegel exercise. Finally, 2 patients in the PKEP group developed urethral stricture 3 months after the operation. However, there were no significant differences in complications between the 2 groups.

**Table 4:** Adverse events in both treatment arms.

	1470 nm laser group (n = 40)	PKEP group (n = 40)	*P* value
Transfusion	1 (2.5%)	3 (7.5%)	.608
Urinary tract infection	1 (2.5%)	2 (5.0%)	1
Urethral stricture	1 (2.5%)	2 (5.0%)	1
Urinary incontinence	0	2 (5.0%)	.494

PKEP = plasma kinetic enucleation of prostate.

## 4. Discussion

In this study, the efficacy of transurethral 1470 nm diode laser vaporization and PKEP for BPH management was compared for the first time. Our findings demonstrated that both procedures are equally effective as surgical approaches, as evidenced by significant improvements in postoperative IPSS, QOL, Qmax, and PVR compared to preoperative assessments. However, 1470 nm diode laser vaporization was superior to PKEP in terms of shortening operation time and reducing intraoperative bleeding. And the results proved the safety and advanced nature of transurethral 1470 nm diode laser vaporization.

Herein, there were no significant differences between the 2 groups prior to surgical intervention, indicating that the baseline conditions of the patients were similar before undergoing either transurethral 1470 nm diode laser vaporization or PKEP. Both procedures resulted in significant improvements in IPSS, QoL, Qmax and PVR at the 24-month follow-up, consistent with previous studies involving transurethral 1470 nm diode laser vaporization^[17,18]^ and PKEP.^[19–22]^

Transurethral 1470 nm diode laser vaporization was associated with significantly less intraoperative blood loss. Indeed, the effectiveness of the laser in vaporizing, cutting and coagulating prostate tissue primarily depends on the laser wavelength. The 1470 nm laser, a new type of diode noncontact laser, generates laser rays through a diode with a penetration depth of 1.30 mm.^[23]^ This laser is selectively absorbed by hemoglobin and water, providing excellent tissue ablation and hemostasis capabilities.^[18]^ Compared with the traditional 1.06 μm YAG laser, the 1470 nm diode laser exhibits stronger tissue absorptivity. Compared with the 10.6 μm CO_2_ laser, the 1470 nm diode laser demonstrates higher cutting efficiency.^[24]^ 1470 nm diode laser not only has the advantages of coagulation and hemostasis from 1.06 μm YAG laser, but also has the advantages of high cutting efficiency from 10.6 μm CO_2_ laser. Besides, the use of optical fiber for guidance during clinical procedures enhances the convenience and safety of employing the 1470 nm diode laser. Seitz et al^[25]^ first reported positive outcomes while using the 1470 nm laser in prostate vaporization surgery, including advantages such as simple operation, a short learning curve, reduced intraoperative bleeding, and rapid postoperative recovery. Subsequently, other scholars have also corroborated these findings, further emphasizing the benefits of semiconductor laser vaporization of the prostate, such as lower intraoperative blood loss and fewer complications.^[26]^

Importantly, the present study found that the operation time was significantly shorter in the 1470 nm diode laser vaporization treatment arm compared to PKEP. This reduction in operation time can be attributed to 2 main factors. Firstly, in recent years, the average power of 1470 nm laser has increased, and independently developed lasers in China has reached 150W. This higher laser power allows for more effective tissue vaporization, leading to a significant reduction in the time required for the procedure. Secondly, the PKEP procedure took significantly more time compared to 1470 nm diode laser vaporization due to the use of a prostatic tissue morcellator to excise large lobe tissue masses.

Herein, we observed a tendency towards a higher rate of transient incontinence following PKEP (5%) compared to 1470 nm diode laser vaporization (0%), although this was not statistically significant It is crucial to emphasize the significance of the prostate’s apex during PKEP procedures, particularly for large prostates, to minimize complications and injuries of the external sphincter during enucleation.

Each surgical BPH treatment varies in the extent of trauma inflicted on the body, leading to different postoperative rehabilitation requirements for patients. Transurethral 1470 nm semiconductor laser prostate enucleation has proven to be effective, but it is essential to consider the potential increase in intraoperative fluid, especially in patients with cardiopulmonary insufficiency, highlighting the need for strengthening hemodynamic monitoring.^[27]^ Bipolar transurethral enucleation of the prostate has shown effectiveness in minimizing the occurrence of the transurethral resection syndrome. Moreover, Bipolar transurethral enucleation of the prostate allows for the resection of more prostate tissues compared to bipolar-TURP, particularly in cases with larger prostates exceeding 80 g.^[28]^ We can specify surgical options for different constitution and the size of the prostate.

The major limitations of our results are rooted in the retrospective, non-randomized matched-controlled study design. Additionally, the 1470 nm diode laser vaporization and PKEP procedures were performed by various surgeons with varying levels of experience. To minimize the impact of surgical expertise on preoperative complications and surgical outcomes, a randomized controlled trial comparing 1470 nm diode laser vaporization to PKEP is warranted in the future.

## 5. Conclusion

Although there are some differences between the 2 procedures both 1470 nm diode laser vaporization and PKEP are effective treatments for BPH. Nevertheless, 1470 nm diode laser vaporization is superior to PKEP in terms of shortening operation time and reducing intraoperative blood loss. However, studies with a larger sample size are needed to further validate our findings.

## Acknowledgments

We would like to thank all the patients who participated in this study. We thank Home for Researchers editorial team (www.home-for-researchers.com) for language editing service.

## Author contributions

**Conceptualization:** Jiaguo Huang, Yi Fan, Kai Wang.

**Data curation:** Jiaguo Huang, Yi Fan, Hongxiang Ding, Dikai Mao, Shengcheng Tai.

**Formal analysis:** Jiaguo Huang, Yi Fan, Liwei Zhao.

**Funding acquisition:** Yi Fan.

**Supervision:** Shengcheng Tai.

**Writing – original draft:** Jiaguo Huang, Yi Fan, Kai Wang, Hongxiang Ding, Dikai Mao, Liwei Zhao.

**Writing – review & editing:** Shengcheng Tai.
